# Two new species of *Tynanthus* Miers (Bignonieae, Bignoniaceae) from Brazil

**DOI:** 10.3897/phytokeys.42.8210

**Published:** 2014-10-24

**Authors:** Maria Cláudia M. P. de Medeiros, Lúcia G. Lohmann

**Affiliations:** 1 Universidade de São Paulo, Instituto de Biociências, Departamento de Botânica, Rua do Matão, 277, 05508-090, São Paulo, SP, Brazil

**Keywords:** Amazonia, Atlantic forest, “cipó-cravo”, lianas, neotropical flora

## Abstract

*Tynanthus* is a genus of lianas that is broadly distributed through the Neotropics. Two new species of *Tynanthus* from Brazil are here described and illustrated: *Tynanthus densiflorus*, from Amazonas, and *Tynanthus espiritosantensis*, from Espírito Santo. *Tynanthus densiflorus* is recognized by the conspicuous interpetiolar glandular fields, a feature rarely found in *Tynanthus*, and the dense thyrses. *Tynanthus espiritosantensis*, on the other hand, is recognized by the bromeliad-like prophylls of the axillary buds and the lax thyrses. Information on the distribution, conservation status and morphologically similar species are provided.

## Introduction

*Tynanthus* Miers (Bignonieae, Bignoniaceae) is a monophyletic genus of lianas that is easily recognized by small bilabiate flowers, fruits with raised margins and by the smell of cloves in vegetative organs (Lohmann 2006). These features, along with flowers arranged in thyrses, corolla externally densely pubescent, thecae curved forward, ovary densely pubescent and poorly developed nectar disk characterize the genus (Medeiros and Lohmann submitted). Species of *Tynanthus* are distributed throughout the Neotropics, occurring predominantly in wet forests (Lohmann and Taylor 2014). The highest diversity of *Tynanthus* is found in Brazil, with most species occurring in Amazonia and the Atlantic Forest.

During the preparation of a taxonomic revision of *Tynanthus* (Medeiros and Lohmann submitted), multiple collections with morphological features that did not match any of the described species were found. Additional fieldwork and molecular phylogenetic studies provided further support for the recognition of two new species in the genus, one from the Amazon region and another from the Atlantic Forest of Brazil.

## Material and methods

Morphological analyses and compilation of phenology and distribution data were based on specimens deposited at CVRD, G, INPA, K, MBM, MG, MO, NY, RB, SP, SPF and US (Thiers, continuously updated). Descriptions were elaborated following terminology presented in Radford (1986), Weberling (1989), the Leaf Architecture Working Group (1999), Gomes-Silva (2009), Nogueira et al. (2013) and Lohmann and Taylor (2014).

### Key to the species of *Tynanthus* with known occurrences in Brazil

**Table:** 

1	Interpetiolar glands present	**2**
1’	Interpetiolar glands absent	**3**
2	Leaflets with caudate-mucronate apices; flowers arranged in dense thyrses	***Tynanthus densiflorus***
2’	Leaflets with acuminate or obtuse-mucronate apices; flowers arranged in lax thyrses	***Tynanthus pubescens***
3	Prophylls of the axillary buds foliaceous	**4**
3’	Prophylls of the axillary buds minute and triangular, or bromeliad-like	**5**
4	Young branchlets puberulent to glabrescent; tendrils trifid; corolla 1.2–1.7 cm	***Tynanthus panurensis***
4’	Young branchlets villous to pubescent; tendrils simple; corolla 0.4–0.8 cm	***Tynanthus polyanthus***
5	Prophylls of the axillary buds minute and triangular	**6**
5’	Prophylls of the axillary buds bromeliad-like	**7**
6	Flowers arranged in dense thyrses; calyx laciniate	***Tynanthus fasciculatus***
6’	Flowers arranged in lax thyrses; calyx minutely denticulate or truncate	**8**
7	Leaflet domatia with trichomes; petioles, petiolules and inflorescence axis without patelliform trichomes	***Tynanthus espiritosantensis***
7’	Leaflet domatia without trichomes; petioles, petiolules and inflorescence axis with patelliform trichomes	***Tynanthus schumannianus***
8	Branchlets tomentose to pubescent throughout; fruits unwinged, with margins slightly raised	***Tynanthus cognatus***
8’	Branchlets glabrescent (sometimes pubescent only at the nodes); fruits winged, with margins prominently raised	**9**
9	Leaflets with acuminate-mucronate apices; corolla 1–1.4 cm	***Tynanthus labiatus***
9’	Leaflets with caudate-mucronate apices; corolla 0.5–0.9 cm	***Tynanthus micranthus***

## Taxonomy

### 1. Tynanthus densiflorus

Taxon classificationPlantaeTubifloraeBignoniaceae

M.C. Medeiros & L.G. Lohmann sp. nov.

urn:lsid:ipni.org:names:77142875-1

#### Type.

**Figure 1.**
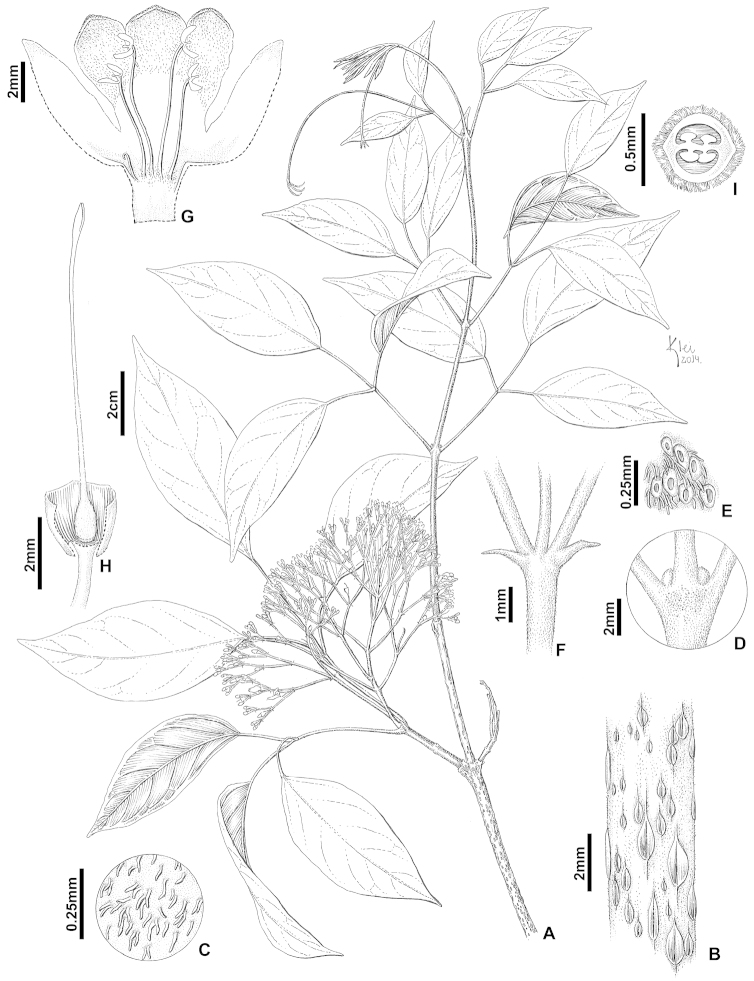
*Tynanthus densiflorus* sp. nov.: **A** Flowering branch **B** Detail of lenticels in the oldest portion of branchelet **C** Detail of pubescent indumentum in the youngest portion of branchelet **D–E** Interpetiolar glands **F** Detail of inflorescence axis with bracts **G** Open corolla showing the androecium **H** Open calyx showing the gynoecium **I** Ovary cross section showing ovules [L.C. Procópio 14 (NY)].

Brazil. Amazonas: Manaus-Itacoatiara, km 26, Reserva Florestal Adolpho Ducke, 16 Aug 1996, L.C. Procópio et al. 14 (holotype: INPA-189631!; isotypes: G!, K!, MG, MO!, NY!, RB!, SP!, U) Fig. 1A–I.

#### Diagnosis.

*Tynanthus densiflorus* differs from *Tynanthus panurensis* (Bureau) Sandwith by the interpetiolar gland fields (lacking in *Tynanthus panurensis*), minute triangular prophylls of the axillary buds (versus foliaceous in *Tynanthus panurensis*) and dense thyrses (versus lax in *Tynanthus panurensis*). It further differs from *Tynanthus pubescens* A.H. Gentry in the leaflets with a caudate-mucronate apex (versus acuminate or obtuse-mucronate apices in *Tynanthus pubescens*) and the dense inflorescences (versus lax in *Tynanthus pubescens*).

#### Description.

*Liana*. *Branchlets* subtetragonal to terete, finely striate, with lenticels, pubescent to puberulent, with simple and peltate trichomes; interpetiolar ridge absent or present; interpetiolar glands present; prophylls of the axillary buds 0.5–0.8 mm long, 1–2.5 mm wide, minute, shallowly triangular, puberulent throughout, with simple and peltate trichomes. *Leaves* 2–3 foliolate; terminal leaflets modified into a trifid tendril; petioles and petiolules with a more or less conspicuous canalicule on the upper side, puberulent to glabrescent throughout, with simple and peltate trichomes; petioles 1.8–5.6 cm long; petiolules (0.6–)1.4–3.8 cm long, lateral ones with equal lengths and the terminal one longer, when present; leaflets (3.2–)5–16.1 cm long, (1.3–)2–9.5 cm wide, membranous to chartaceous (sometimes subcoriaceous), discolor or concolor, ovate, apex caudate, mucronate, base cuneate to truncate or subcordate, symmetrical or asymmetrical, margin entire; the abaxial surface pubescent to puberulent throughout (sometimes only on and near the veins), with simple, peltate and patelliform trichomes; the adaxial surface pubescent to glabrescent throughout (sometimes only on and near the veins), with simple, peltate and patelliform trichomes; glandular trichomes evenly distributed throughout both surfaces; first venation pinnate, second venation weak brochidodromous, third venation alternate percurrent (sometimes random reticulate); pocket domatia with (sometimes without) trichomes. *Inflorescence* 3–9.5 cm long, a thyrse, axillary, dense, corymbose to conical in aspect; axis densely pubescent to puberulent, with simple and peltate trichomes; inflorescence bracts 0.5–2.5 mm long, predominantly caducous, triangular to linear triangular, densely pubescent to pubescent throughout; floral bracts 0.4–0.6 mm long, triangular; floral pedicels 1–7 mm long. *Calyx* green to grayish, 1.5–2.2 mm long, 1.4–1.9 mm wide, membranous to chartaceous, with a transversal aperture, truncate or minutely 5-denticulate, densely pubescent to pubescent outside, with simple and peltate trichomes, glabrous inside; lobes 0.1–0.2 mm long. *Corolla* cream or pale yellow, 0.8–1.5 cm long, 0.3–0.5 cm wide at the tube opening, bilabiate, with two (almost totally fused) upper lobes and three lower lobes, densely pubescent throughout outside, with simple and peltate trichomes; tube 3–5 mm long, internally glabrous at the top, tomentose at the base, with simple, long and short stipitate trichomes; nectar guides present, yellow; lobes entire, densely pubescent to pubescent throughout lower ones and at margins of or throughout upper ones; upper ones 0.4–1.4(–2.9) mm long, 0.7–1.5(–2.4) mm wide, acute to obtuse; lower ones 2.1–4 mm long, 2–3.6 mm wide, obtuse to rounded. *Androecium* with four fertile stamens inserted at 1.5–2.5 mm from the base of the corolla; shorter ones 3.5–5.5 mm long; longer ones 4.5–7 mm long; filaments with long and short stipitate trichomes at the base; anther thecae cream, 1.1–1.4 mm long, obovate to elliptic, divergent and reflexed forward, glabrous, subexserted; connective extending 0.2–0.3 mm beyond anther attachment; staminode covered with long and short stipitate trichomes, 1.5–2.7 mm long. *Gynoecium* ca. 7–9 mm long; ovary 1.3–1.5 mm long, 0.7–0.8 mm wide, conical, velutinous, with simple trichomes, with a ring of longer trichomes at the base, with two or four series of ovules per locule; nectar disc reduced, not evident; style 5–7 mm long, tomentose at the base, with simple trichomes; stigma with lamellae lanceolate, glabrous. *Fruit* not seen. *Seeds* not seen.

#### Distribution and habitat.

Known from wet forests in Manaus and proximity (Amazonas, Brazil).

#### Etymology.

The species epithet refers to the thyrses with flowers densely arranged.

#### Phenology.

Flowers in August. Fruiting period is unknown.

#### Conservation status.

According to IUCN (2001) criteria, this species is considered Vulnerable (VU B2ab(iii)). The type collection is from a protected area (Reserva Florestal Adolpho Ducke), where a reasonable number of individuals are found (pers. obs.). Nevertheless, the proximity of Manaus can be considered a region characterized by continuous urbanization. Additional studies on distribution and abundance of *Tynanthus densiflorus* are still necessary in order to confirm its conservation status.

#### Discussion.

*Tynanthus densiflorus* is characterized by dense thyrses, with a corymbose to conical aspect, as well as the presence of interpetiolar glands. The type collection of *Tynanthus densiflorus* was treated as *Tynanthus panurensis* (Bureau) Sandwith for the Guide of the Ducke Reserve (Lohmann and Hopkins 1999). Indeed, the two species are similar in the occurrence of ovate leaflets and corolla tube that is internally tomentose at base. However, the presence of interpetiolar glands in *Tynanthus densiflorus* (versus absent in *Tynanthus panurensis*), the minute prophylls (versus foliaceous in *Tynanthus panurensis*) and dense inflorescences (versus lax in *Tynanthus panurensis*) allow the distinction of these taxa. *Tynanthus pubescens* A.H. Gentry is another species that is morphologically similar to *Tynanthus densiflorus*. Both taxa have similar corolla lengths (around 1–1.6 cm in *Tynanthus pubescens*) and show interpetiolar gland fields; however, the caudate-mucronate leaflet apex (versus acuminate or obtuse-mucronate in *Tynanthus pubescens*) and the dense inflorescences (versus lax in *Tynanthus pubescens*) differentiate both taxa.

#### Additional specimens examined.

BRAZIL. Amazonas: 2-5 km N of Manaus-Itacoatiara Road at km 79 near Rio Preto da Eva, 100–200 m, 24 November 1974, *A. Gentry 12849* (INPA, MG, MO). Rio Camanau, 28 June 1987, *P. Grenand et al. 2787* (INPA). Manaus, Campus of INPA, Estrada do Aleixo, 22 November 1974, *A. Gentry 12792* (INPA); 30 November 1974, *A. Gentry 13018* (INPA, MO); *Ibid.*, Transect vouchers, Line 1, 11 December 1974, *A. Gentry 13181* (INPA, MO); Estrada do Aleixo near Manaus, km 6–7 past INPA, 2 December 1974, *A. Gentry 13040* (INPA, MO); Reserva Florestal Adolpho Ducke, Parcela PPBio (L03 1000 m), 100 m, 02°56'03"S, 59°57'32"W, 14 December 2010, *M.C. Medeiros et al. 21* (SPF); *Ibid.*, próximo à estação meteorológica, 120 m, 02°55'37"S, 59°58'33"W, 15 December 2010, *M.C. Medeiros et al. 22* (SPF); *Ibid.*, proximidades do refeitório da base da reserva, na beira da estrada, 110 m, 02°55'59"S, 59°57'56"W, 16 December 2010, *M.C. Medeiros et al. 25* (SPF).

### 2. Tynanthus espiritosantensis

Taxon classificationPlantaeTubifloraeBignoniaceae

M.C. Medeiros & L.G. Lohmann sp. nov.

urn:lsid:ipni.org:names:77142876-1

#### Type.

**Figure 2.**
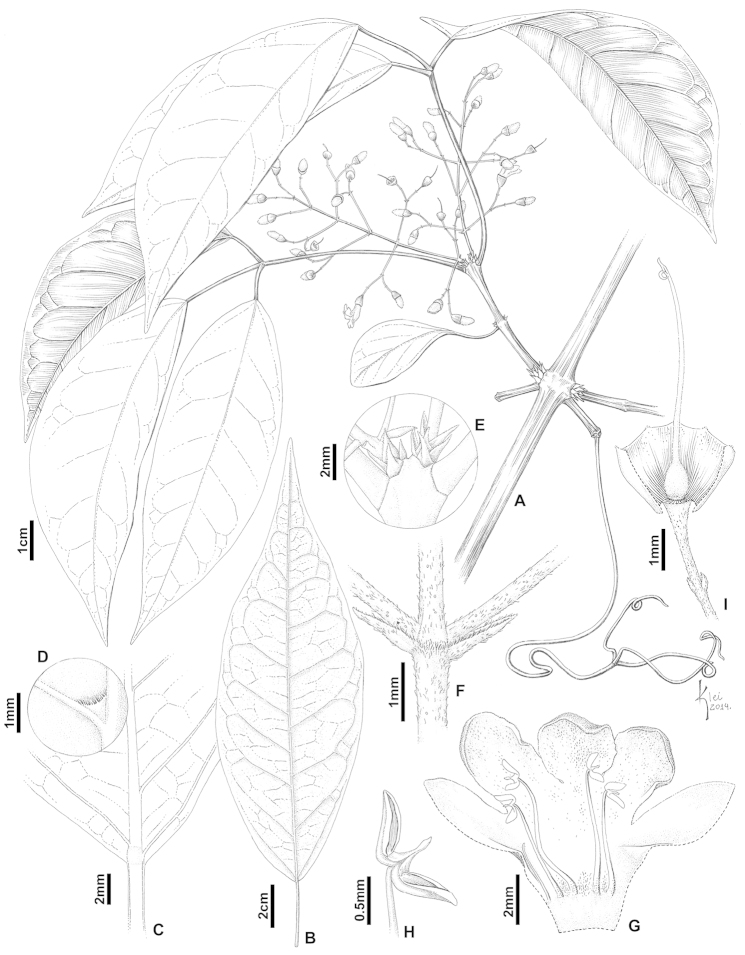
*Tynanthus espiritosantensis* sp. nov.: **A** Flowering branch **B–D** Leaflet with pubescent domatia in the abaxial surface **E** Interpetiolar region with bromeliad-like prophylls of the axillary buds **F** Detail the of inflorescence axis, showing bracts, simple and peltate trichomes **G** Open corolla, showing the androecium **H** Anther **I** Open calyx showing the gynoecium [D.A. Folli 5931 (SPF)].

Brazil. Espírito Santo: Linhares, Reserva Natural da CVRD, Estrada Oiticica, km 2.3, 6 Feb 2008, D.A. Folli 5931 (holotype: CVRD-11073!; isotype: SPF!) Fig. 2A–I.

#### Diagnosis.

*Tynanthus espiritosantensis* differs from *Tynanthus schumannianus* (Kuntze) A.H. Gentry by the presence of trichomes in the leaflet domatia (versus absence in *Tynanthus schumannianus*), lack of patelliform trichomes on petioles, petiolules and inflorescence axis (versus presence in *Tynanthus schumannianus*) and larger calyx, 2.3–2.7 mm long, 1.8–2.5 mm wide (versus 1–2 mm long, 1.1–1.9 mm wide in *Tynanthus schumannianus*).

#### Description.

*Liana*. *Branchlets* tetragonal to terete, finely striate, with lenticels, glabrescent (sometimes pubescent at the nodes), with peltate and patelliform trichomes (sometimes simple trichomes also present); interpetiolar ridge absent; interpetiolar glands absent; prophylls of the axillary buds 1.2–2.5 mm long, 0.7–1.1 mm wide, bromeliad-like, glabrescent (rarely puberulent), with peltate trichomes (rarely with simple trichomes as well). *Leaves* (2–)3 foliolate; terminal leaflets modified into a trifid tendril; petioles and petiolules with a more or less conspicuous canalicule on the upper side, puberulent throughout, with simple and peltate trichomes; petioles 1–6 cm long; petiolules 0.5–3.5 cm long, lateral ones with equal lengths and the terminal one longer, when present; leaflets (4–)5–11.9 cm long, (1.5–)1.9–5.4 cm wide, membranous to chartaceous, discolor, elliptic, apex acuminate or caudate, mucronate, base cuneate, symmetrical, margin entire; the abaxial surface glabrescent (sometimes pubescent) on and near the veins, with peltate and patelliform trichomes (sometimes also simple); the adaxial surface glabrescent on and near the veins, with peltate and patelliform trichomes; glandular trichomes distributed especially on the abaxial surface; first venation pinnate, second venation weak brochidodromous, third venation alternate percurrent (sometimes random reticulate); pocket domatia with trichomes. *Inflorescence* 3.6–7 cm long, a thyrse, axillary, lax, conical in aspect; axis pubescent, with simple and peltate trichomes; inflorescence bracts 0.7–3.9(–9) mm long, predominantly caducous, triangular to linear triangular, pubescent throughout or only at margins; floral bracts 0.5–0.7 mm long, triangular; floral pedicels 3.5–9 mm long. *Calyx* green, 2.3–2.7 mm long, 1.8–2.5 mm wide, membranous to chartaceous, with a transversal (sometimes oblique) aperture, minutely 5-denticulate, glabrescent (sometimes pubescent at teeth) outside, with simple, peltate and patelliform trichomes, glabrous inside; lobes 0.1–0.4 mm long. *Corolla* white, 0.7–0.8 cm long, 0.25–0.34 cm wide at the tube opening, bilabiate, with two (almost totally fused) upper lobes and three lower lobes, densely pubescent throughout outside, with simple and peltate trichomes; tube 2.5–4 mm long, internally glabrous at the top, tomentose to pubescent at the base or glabrescent, with simple, long and short stipitate trichomes; nectar guides absent, but with a path of long and short stipitate trichomes; lobes entire, densely pubescent to pubescent throughout lower ones and at the margin of upper ones; upper ones 0.4–1.1 mm long, 1–1.5 mm wide, acute to obtuse; lower ones 1.8–3.2 mm long, 2.1–2.5 mm wide, obtuse to rounded (sometimes acute). *Androecium* with four fertile stamens, inserted at 1–1.5 mm from the base of the corolla; shorter ones 2.5–3.5 mm long; longer ones 4.5–5 mm long; filaments with long and short stipitate trichomes at the base; anthers thecae cream, 0.8–1.1 mm long, obovate to elliptic, divergent and reflexed forward, glabrous, subexserted; conective extending 0.2–0.3 mm beyond anther attachment; staminode glabrescent, with long and short stipitate trichomes, 2.4 mm long. *Gynoecium* ca. 4.5–6 mm long; ovary 0.8–1 mm long, 0.7–0.9 mm wide, conical, velutinous, with simple trichomes, with a ring of longer trichomes at the base, with two or four series of ovules per locule; nectar disc reduced, not evident; style 3.3–5 mm long, tomentose at the base, with simple trichomes; stigma with lamellae lanceolate, glabrous. *Fruits* not seen. *Seeds* not seen.

#### Distribution and habitat.

Known exclusively from wet forests in Linhares and proximity (Espírito Santo, Brazil).

#### Etymology.

The species epithet refers to the type locality.

#### Phenology.

Flowers from December to February. Fruiting period is unknown.

#### Conservation status.

According to the IUCN (2001) criteria, this species is considered Data Deficient (DD), given the small number of known collections. Further detailed investigation on the distribution of *Tynanthus espiritosantensis* is necessary in order to properly assess its conservation status. The two localities in which this species has been collected fall within a single municipality (Linhares), suggesting that this might represent another narrowly distributed species of *Tynanthus*. Fortunately, the type collection was obtained inside a protected area (Reserva Natural da CVRD).

#### Discussion.

*Tynanthus espiritosantensis* is characterized by the bromeliad-like prophylls of the axillary buds and lax thyrses. This species is morphologically similar to the Amazonian *Tynanthus schumannianus* (Kuntze) A.H. Gentry. However, these taxa can be easily separated by the pubescent leaflet domatia (versus glabrous in *Tynanthus schumannianus*), absence of patelliform glands on petioles, petiolules and inflorescence axis (versus presence in *Tynanthus schumannianus*) and the larger calyx, 2.3–2.7 mm long, 1.8–2.5 mm wide (versus 1–2 mm long, 1.1–1.9 mm wide in *Tynanthus schumannianus*).

#### Additional specimens examined.

BRAZIL. Espírito Santo: Linhares, Rancho Alto, 7 December 1984, *G. Hatschbach & J.M. Silva 48693* (MBM, MO, US); Reserva Natural da CVRD, Estrada Oiticica, próximo à porteira, antes do cruzamento com a estrada municipal, 53 m, 19°07'59"S, 40°00'07"W, 27 January 2014, *M.C. Medeiros & R.B. Louzada 41* (CVRD, SPF).

## Supplementary Material

XML Treatment for Tynanthus densiflorus

XML Treatment for Tynanthus espiritosantensis
